# Encouraging outpatients in an acute hospital for the relief of cancer-related pain: a qualitative study

**DOI:** 10.1186/s12904-023-01236-y

**Published:** 2023-08-01

**Authors:** Miwa Hinata, Kikuko Miyazaki, Takeo Nakayama, Megumi Tokunaga, Toru Watanabe, Shuichi Nawata

**Affiliations:** 1 Department of Hospital Pharmaceutics, School of Pharmacy, Showa University, 1-5-8 Hatanodai Shinagawa-ku, Tokyo, 142-8555 Japan; 2 Department of Health Informatics, Kyoto University School of Public Health, Kyoto, Japan

**Keywords:** Pain, cancer, Outpatients, Medical, Hospitals, Qualitative research

## Abstract

**Purpose:**

To identify the processes of cancer-related pain relief and exacerbation faced by outpatients in an acute care hospital and to examine the support needed for outpatient pain control.

**Methods:**

We conducted semi-structured, in-depth interviews with patients from the outpatient department of Showa University Northern Yokohama Hospital in Kanagawa Prefecture, Japan. Participants were recruited by purposive sampling. From the recorded data, verbatim transcripts were made and used as textual data for analysis by consistent comparative method.

**Results:**

Between April 2018 and April 2022, interviews were conducted with 30 participants. Analysis of the verbatim transcripts generated 13 categories from 27 concepts. Category relationships were examined, and a conceptual framework was developed. Outpatients went from being in a state of hesitation towards consultation with medical professionals to receiving individual consistent follow-ups by medical professionals in the hospital and community pharmacies, which led to patient teleconsultations when their physical condition changed, leading to an improvement of pain.

**Conclusion:**

The process of relief and exacerbation of cancer-related pain experienced by outpatients in the acute care hospital reveals that the provision of consistent follow-up through remote or in-person interviews has an important role to play in pain management, as it helps to build relationships between patients and medical professionals. Alternatively, when outpatients exhibited endurance, their pain worsened, and they fell into a negative cycle of poor pain control.

## Introduction

To meet the needs of all patients, different levels of palliative care have recently been proposed: primary, secondary, and tertiary [1]. Palliative care provided by hospital oncologists is considered secondary, for which there are no formal practice guidelines [2], and a need for specialist education has been identified [3]. There are also significant barriers to implementing the transition to palliative care in acute care hospitals [4]. In Japan, less than 5% of cancer practice guidelines include palliative care [5], and there are challenges in integrating oncology and palliative care.

An increasing number of outpatients have undergone cancer drug treatment in recent years [6]. It is estimated that more than 65% of patients experience cancer-related pain [7]. A survey of Japanese outpatients undergoing cancer treatment found that 20% of the patients experienced moderate to severe pain [8]. In Japan, only half of hospitals routinely screen outpatients for symptoms [9], and a national survey found that 43% of patients reported that the care to alleviate physical pain was adequate [10]. While cross-sectional surveys are beginning to identify the physical and emotional problems faced by outpatients undergoing cancer-related pain management [8, 10, 11], the factors that influence pain relief in outpatients are not known. We aimed to identify the processes of cancer-related pain relief and exacerbation faced by outpatients in an acute care hospital and to examine the support needed for outpatient pain control.

## Methods

We conducted a qualitative semi-structured, in-depth interviews with patients from the outpatient department of Showa University Northern Yokohama Hospital in Kanagawa Prefecture, Japan. An interview guide was developed with the consensus of hospital and community pharmacists (Table 1). The study was conducted as a one-to-one, in-depth interview to determine the true feelings of outpatients regarding the barriers to pain control. The interviewer had at least one or more meetings to assess the patient’s status and build rapport before the interview. The interviews, recorded with informed patient consent, were conducted in the wards or common rooms, outpatient waiting rooms, and outpatient chemotherapy rooms.

The first author (MH), a female hospital pharmacist trained by a specialist, conducted the interviews. Age (date of birth), gender, primary disease, chemotherapy, and medications were collected from the medical records of the consenting study participants. Eastern Cooperative Oncology Group (ECOG) Performance Status were recorded by the researcher, as determined during the interviews. Field notes were made for each survey, describing the participants and their situation at the time of the survey.

**Table 1:** Interview guide*

Topic	Question
Pain management	What do you do if you are in a lot of pain and have problems at home?
Rescue	Did you take pain relief (rescue) medications without hesitation?
Explanation	Was the medication fully explained to you? Did you understand it well?
Adverse effects	Have you had any problems with adverse events? How did you cope with the adverse events?
Communication	Was communication with the doctor/pharmacist sufficient?
Family influences	Whether the family’s opinion had a significant influence on the treatment.

*Based on the agreement between hospital pharmacists and community pharmacists.

### Selection of participants

Participants were recruited by purposive sampling. Selection criteria were: outpatients with advanced cancer, who had been administered medical narcotics for cancer pain control at Showa University Northern Yokohama Hospital; patients aged 20–80 years at the time of consent; and patients who provided written informed consent to participate in the study. Exclusion criteria were patients with a Numerical Rating Scale pain scale score ≥ 3 at rest to avoid interviewing patients in severe pain, patients who were judged ineligible by the doctor in charge, and patients who were unable to communicate in Japanese. The study was approved by the research ethics committee of Showa University Northern Yokohama Hospital (31 June 2018; approval number:17H097). The purpose of the study and protection of personal privacy were explained to the participants and written informed consent.

### Data analysis

From the recorded data, verbatim transcripts were created and used as textual data for analysis by constant comparative analysis [12, 13], an excellent theory to explain and predict human behavior. The textual data were analysed using Nvivo 1.6.1®ฏ. We initially analysed the data inductively by checking the analysis theme while reviewing the records, and then developed concepts from each part of the focused text (called “variations”), corresponding to the theme. The concept creation and naming process was recorded in theoretical memos. When a concept was near completion, the textual data was examined again to determine if there was an opposing concept. When an opposite case existed, the concept was finalized; if no opposite existed, this was noted in the theoretical memo of the analysis worksheet. Concept development and opposite case-finding were repeated to generate further concepts and categories, and the relationships between the concepts and categories were examined and recorded in theoretical memos. The findings generated through this process were reviewed by a supervisor (KM) and multiple analysts, and their findings triangulated [14].

## Results

Between April 2018 and April 2022, interviews were conducted with participants who met the eligibility criteria. We recruited 32 people, two of whom did not participate further due to not knowing what to say. Theoretical saturation was considered to have been reached when 30 interviews were conducted (14 female and 16 male participants).

The average duration of the interviews was approximately 39 min. We conducted interviews at Hospital room(n = 11), Outpatient chemotherapy room(n = 10), Lounge(n = 7), Waiting room(n = 2). There were five participants in their forties, eight in their fifties, seven in their sixties, and 10 in their seventies or older. Primary diseases of participants included gastrointestinal cancer (n = 5), lung cancer (n = 15), breast cancer (n = 3), urological cancer (n = 4), and other (n = 3). In addition, 22 participants were undergoing chemotherapy and eight were receiving best supportive care (BSC) (Table 2).

**Table 2:** Patient characteristics (n = 30)

Characteristic	n
Gender	
Male	16
Female	14
Age	
40s	5
50s	8
60s	7
70s	9
80s	1
ECOG Performance Status	
0	7
1	22
2	1
Primary site	
Lung, chest	15
Colon, rectum	2
Stomach	2
Uterus, ovary	2
Breast	3
Prostate, ureter, bladder	4
Others	2
Chemotherapy	
Under treatment	22
BSC	8
*Opioid dosage (mg)	
0, ≦30	13
30, ≦100	12
100	5

ECOG, Eastern Cooperative Oncology Group; BSC, best supportive care; Opioid dosage (mg), converted to oral morphine equivalent (mg).

Interviews were conducted while taking into account the physical condition of the participants, with interviews conducted in multiple sessions if necessary. Analysis of the verbatim transcripts generated 13 categories from 27 concepts (Table 3).

**Table 3:** Categories and Concepts

Category	Concept	Definition
Unexpressed pain	Difficulty in communicating pain	Difficulty communicating pain to healthcare professionals and others, because of difficulties in communicating pain, such as different standards for different people, even when trying to convey how painful it is
Resistance to narcotics	Fear of drug addiction	Avoiding taking narcotic rescue as much as possible because of concerns that medical narcotics may have negative effects on the body, such as dependence
	Experiencing the painful adverse event of narcotics	Difficult experience with medical narcotics due to strong side effects such as drowsiness, constipation, nausea, and delirium
Concerns about medication	Hard-to-obtain narcotics	Not being able to get medicines quickly because community pharmacies often do not stock medical narcotics
	Anxiety about side effects	Worrying about the effects on the body of having to take so many different medicines to stop the pain
	Difficulty in rescue opioid timing	Difficulty in deciding when to use rescue and at what intervals
Hesitation towards consultation	Atmosphere of difficulty to consult	Finding it difficult to ask questions of medical professionals about uncertainties regarding treatment and use of medicines
	Exhaustion due to waiting times	Long waiting times at both hospitals and community pharmacies, and the enormous amount of effort required to see a doctor and receive medicines while being unwell
	Considerations for medical professionals	Refraining from asking medical professionals for more information or making requests because the outpatient clinic or community pharmacy seems too busy
Pain endurance	Choosing patience	Choosing to persist at home, even in the presence of severe pain
	Coping by self-judgment	Managing pain and symptoms at home by self-medicating when symptoms or changes in physical condition occur
	Opioid rescue savings	Having to save and take rescue internally to avoid running out of narcotic rescue before the next visit to the doctor
Persistent pain	Untreated pain	Going about their daily life with pain that did not subside, even when treated with medication
	Exacerbation of pain	Increased pain symptoms at home
Anguish over pain	Pessimism about pain	Feeling resigned to pain persisting
	Anxiety about the future	Suffering from a lack of visibility about their illness and what their treatment will look like in the future
	Mental anguish	Undergoing treatment while having mental anguish
Emergency hospitalization	Pain beyond endurance	Emergency hospitalization due to severe and unbearable pain at home
Approach by medical professionals	Medical professionals who always care	The presence of a medical professional who is always caring toward them
	Remote follow-up suggestion	That the medical professional has made a suggestion to follow-up remotely by telephone at home
Consistent follow-up	Remote follow-up reassurance	Being reassured by having a remote check of their condition at home from a medical professional
	Multidisciplinary support	Being supported by multiple professions in pain management
	Multidisciplinary information sharing	Feeling that consultations were shared with many different professions
Teleconsultation	Consultation at home	Being able to consult remotely from home when there was a change in their health condition
	Medical professionals to consult	Having a medical professional to consult when feeling unwell or in need of help
Urgent hospital visit	Encouragement to consult a doctor	Being encouraged to consult a medical professional about changes in their condition at home and to see a doctor
Improvement of pain	Appropriate use of drugs	Improved pain and ability to move with the appropriate use of medication for pain relief

**Fig. 1:**
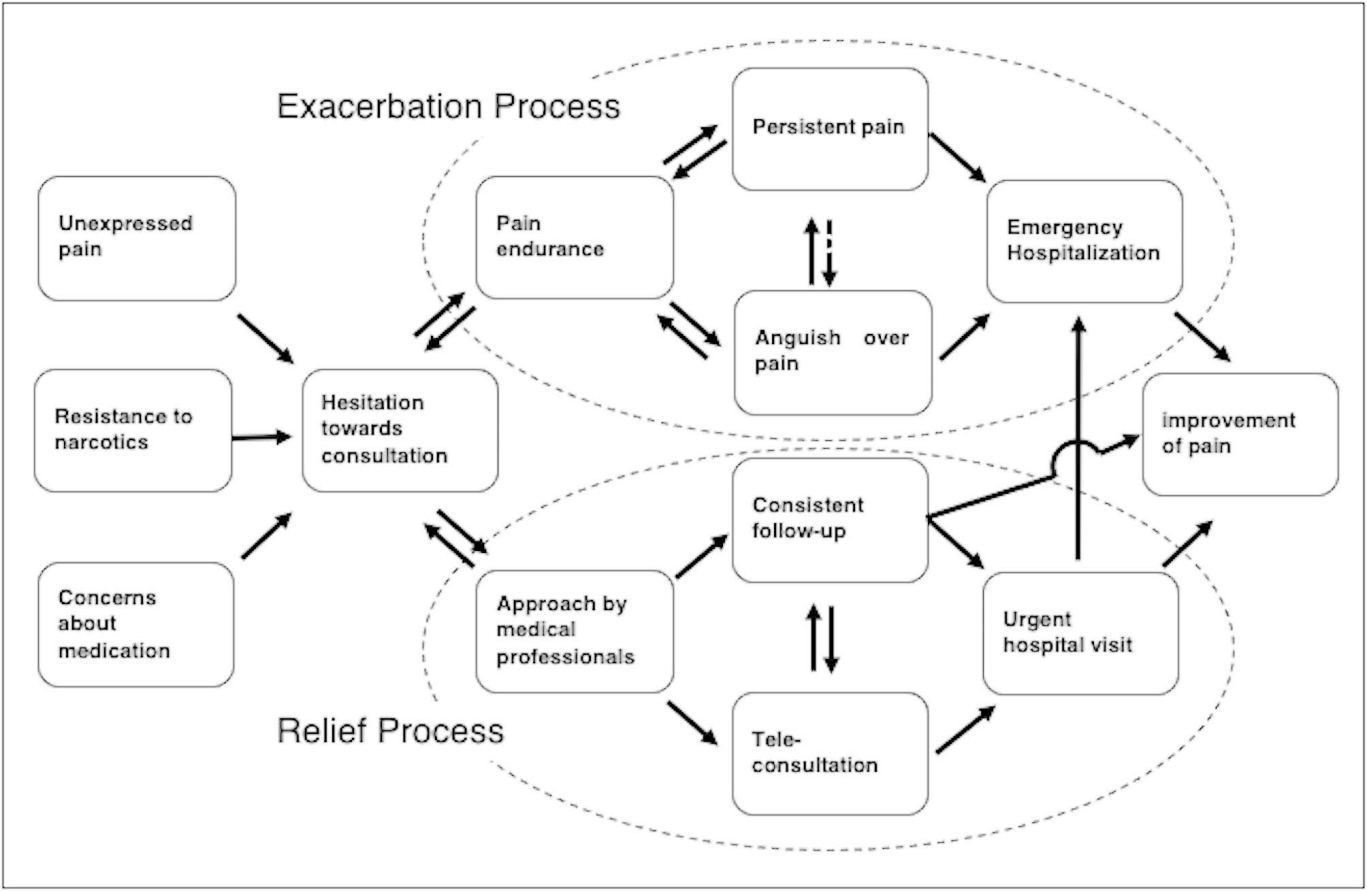
Conceptual framework of the study, showing the relief and exacerbation process of cancer-related pain

Category relationships were examined, and a conceptual framework was developed (Fig. 1).

### Storyline

We constructed a storyline using each participant’s category (indicated in **bold**), concept (indicated in **bold**), and narrative (indicated in *italics*). Outpatients with cancer-related pain control had **unexpressed pain** due to **difficulties in communicating pain**, an example of which was: *“But I think it’s difficult to communicate. Because pain can be different to the person who thinks it’s painful”* (Patient A). Patients also experienced **resistance to narcotics** from the **fear of drug addiction** and **experiencing the painful adverse event of narcotics**: *“That is to forcibly stop the pain, isn’t it? So, in that sense, I think it is not good for us, so we tried to endure as much as we could, but…”* (Patient B). In addition, participants had various **concerns about medication**, such as **anxiety about side effects**, **difficulty in rescue opioid timing**, and **hard-to-obtain narcotics**: *“I used to take very few medicines, but now I take seven or eight in the morning, five or six at lunchtime, nine at night, and one at night. I’m getting used to it, but when I’m living a normal life, I’m not sure if it’s safe to take that many pills. I have a fear that I might get something from the combination”* (Patient C). However, outpatients experienced **hesitation towards consultations** due to **difficulty in the consultation atmosphere**, **consideration for the medical professionals**, and **exhaustion due to waiting times** in hospitals and community pharmacies: *“Generally, when I talk to the pharmacist, I only talk to the pharmacist when I ask for my medication, and they are just going through the motions, giving me the medication, and telling me what the medication is. They don’t know much more about me than that.”* (Patient D).

Outpatients had **pain endurance** through **coping by self-judgment** and **choosing patience** and **opioid rescue savings**, even when they were in pain at home: *“In my mind, I just put up with the pain and told myself that it’s normal for it to feel painful because I have a tumour like this now, or that it’s just something that can’t be helped, so I just put up with it”* (Patient E). However, due to **untreated pain** and **exacerbation of pain**, participants had **persistent pain** in their daily lives: *“Well, I had problems with the medication not working; and, as a result, I think it was because the dose was too low; and, as I said before, I was a patient person, so they didn’t prescribe much medicine. So, when the medication ran out, it was really hard, and I did nothing but fainting in agony.”* (Patient A). Furthermore, outpatients had **anguish over pain** due to **mental anguish**, **pessimism about pain**, and **anxiety about the future**, leading to a negative spiral of pain control: *“So, I’ve already given up halfway, or rather, to the pain, towards the pain, just the pain, just saying, it’s pain here. I don’t think I’m holding back, I think I’m just enduring the pain, the pain that’s right in front of me right now”* (Patient F).

**Pain beyond endurance** made **emergency hospitalization** necessary: *“Generally, if I go there, in my case, they say, ‘Oh, you’re going to be hospitalized.’ But that one step is a bit. If they told me to go home again, I felt like I’d gone all the way there and now I have to go home again with all this pain”* (Patient G). On the other hand, there were **approaches by medical professionals** such as the presence of **medical professionals who always care** and **remote follow-up suggestions** to outpatients: *“When I was getting my medication, the community pharmacist asked me if he could call me at home. And I said, ‘Please.’ So, they set a date for me to contact them and that’s when I got the call”* (Patient H).

In **consistent follow-up**, outpatients felt **multidisciplinary support**, **remote follow-up reassurance**, and **multidisciplinary information sharing**: *“I thought, ‘The fact that they are calling me means that they are worried about me, and I need to do something to respond to them, like keeping a diary or something, instead of being so careless’”* (Patient I).

**Consistent follow-up** led to **teleconsultations**, where outpatients received **consults at home** from **medical professionals**: *“Well, he gave me his mobile phone number, the number to call at midnight, and I left him a message, and the next day he called me on my mobile phone. He said he was sorry he couldn’t reply immediately. I didn’t expect such a quick response. I just needed you to listen to what I had to say”* (Patient J).

When patients were not sure whether they should go to hospital, they had a medical professional **encourage them to consult a doctor** and they were subsequently examined by a doctor through **urgent hospital visits**: *“I called them just to ask about that, whether I should go to the hospital or not. I was told to go to the hospital and was admitted immediately. So, I knew I needed to ask for advice”* (Patient K). Outpatients experienced **improvement of pain** due to the **appropriate use of drugs**: *“Honesty, well, it doesn’t go that far. It’s not that I don’t feel a little bit of some pain relief. There, you know, mentally, it’s a little bit easier than it was two weeks ago”* (Patient L).

## Discussion

The process of relief and exacerbation of cancer-related pain experienced by outpatients in the acute care hospital reveals that the provision of consistent follow-up has an important role to play in pain management, as it helps to build relationships between patients and medical professionals. Outpatients progress from a state of hesitation towards consultation to consistent follow-up owing to the medical professionals’ approach in the hospital or community pharmacy, and when their physical condition changes, patients receive consultations telephonically, leading to improved pain management. Alternatively, when outpatients exhibited endurance, their pain worsened, and they fell into a negative cycle of poor pain control. The patient was then in a vicious circle of untreated pain, pessimism regarding the future, and further psychological anguish over pain. Patients endured at home until they reached the limit of their endurance, and then followed the process of emergency hospitalization.

### Patient behaviour and social cognitive theory

The changes in outpatient behaviour identified in this study can be interpreted using social cognitive theory (SCT). SCT states that human behaviour is always dynamic due to the interaction of three factors: person, environment, and behaviour [15]. We suggest that the patient’s behaviour of hesitation towards consultation was influenced by the approach by medical professionals and consistent follow-up, which changed the environment and affected the person in building a relationship with the medical professionals, leading to the spontaneous behaviour of teleconsultation. On the other hand, when outpatients continued to be hesitant toward consultation and experienced pain endurance, they fell into a vicious circle of anguish over pain. Physical symptoms such as pain and nausea/vomiting are closely related to psychological functioning [16]. Kimura et al. also reported that patients believe it is impossible to talk to doctors and pharmacists on an equal footing due to a lack of medical knowledge, and, therefore, patients tend to simply defer to specialists [17]. Furthermore, in a national survey, approximately 50% of Japanese outpatients with cancer thought that they felt like a burden to others [8]. It can be inferred that this reluctance to leave the matter to specialists and the desire not to be a burden others complicated the person and prevented patients from acting on their own, leading to a shift from hesitation towards consultation to pain endurance.

### Outpatient environment and the process of pain exacerbation

Based on SCT [15], the patient behaviour of hesitation towards consultation and pain endurance are considered to be the result of the person being influenced by the environment. In Japan, the proportion of separate dispensing and prescribing functions is over 70% [18], and patients go to the community pharmacy with the prescription issued by the doctor. Information sharing between hospital and pharmacy about outpatients is rarely done, with pharmacists providing medication guidance based solely on information elicited from patients [19]. Lack of effective communication between medical professionals and patients in acute care hospitals may contribute to difficulties in transitioning to palliative care [4].

Early provision of palliative care reduces unnecessary hospital admissions and use of health services [20]. On the other hand, despite the effective value of palliative care, significant barriers remain in terms of integrating palliative care services into the existing healthcare system [21]. It is generally difficult in Japan to share treatment plans for individual outpatients, and it is difficult to intervene in a multidisciplinary fashion regarding the management of patient pain. Hesitation towards consultation in outpatients may have been influenced by this background. This suggests that there is an urgent need to address the environment on the part of medical professionals to prevent patients from engaging in pain endurance.

### Consistent follow-up and the process of pain relief

Those patients who received consistent follow-up were able to improve pain without having to wait for the endurance limit through teleconsultation when their pain worsened. Palliative care for outpatients in acute care hospitals is positioned as secondary palliative care, where cancer specialists provide pain management alongside cancer treatment. None of the participants-attending physicians in this study specialized in palliative care. The approach by medical professionals and subsequent consistent follow-up by doctors, nurses, and hospital and community pharmacists who do not specialize in palliative care may function as a type of patient education and contribute to improving patient pain. A systematic review of randomized controlled trials on pain management found that patient education resulted in a statistically significant reduction in pain intensity [22]. Patients are anxious about medicines and their potential side effects [17]. In this study, the narrative was also that patients who received consistent remote follow-up were able to use the service effectively, asking questions about their treatment and how to use the medication, as well as other matters they were anxious about while using the medication at home. Patient education programs that have proven effective so far have included a coaching component to address individual patient concerns, rather than just providing pain-related material [23–26]. However, in the model of teleconsultation in supply-driven palliative care, excessive attention to symptoms and potential distress has been reported to worsen symptom scores among home-based patients with advanced cancer [27]. The frequency and timing of appropriate consistent follow-up should be considered, in line with the convenience on the part of outpatients.

### Palliative care issues for outpatient settings

Our findings suggest that the relationship established between medical professionals and patients through consistent follow-up has an important role in pain management, as it succeeds in eliciting active consultation from patients. However, consistent follow-up of outpatients by medical professionals through remote or in-person interviews is an initiative that has been implemented in hospitals and community pharmacies for some patients with pain control problems, and there is no mechanism to calculate a medical service fee. Consistent follow-up by medical professionals through the telephone or PC should be implemented for all patients with pain management as part of the palliative care service for outpatients. Furthermore, if community pharmacists conduct consistent follow-up, the subsequent sharing of information to the hospital is important. Previous studies have shown the importance of team involvement in palliative care for cancer patients from an early stage [28]. The community pharmacist who conducted consistent follow-up for the patients in this study shared the information with the hospital using a tracing report, and the hospital staff responded at the next visit, if necessary. For pain management in outpatients, further studies should focus on how to share appropriate information between community pharmacies and hospitals, and how to select patients who should be consistently followed-up.

### Study limitations

The study recruited subjects from only one acute hospital, which may differ from other hospitals and community pharmacy initiatives. The unique circumstances of this study need to be carefully considered before they can be applied to the palliative care of outpatients with cancer in other settings. Sampling was done by purposive sampling and at least one patient was recruited from all departments treating cancer to prevent bias in patient opinion, but selection bias due to patient physical condition considerations and the fact that the investigator was a hospital pharmacist meant that critical opinions may not have been adequately elicited. However, the processes of cancer-related pain relief and exacerbation faced by outpatients in an acute care hospital in the context of secondary palliative care, as identified in this study, focus on the psychological aspects of outpatients. Therefore, we believe that it can be applied in other regions as well.

## Conclusions

The process of relief and exacerbation of cancer-related pain experienced by outpatients in the acute care hospital reveals that the provision of consistent follow-up through remote or in-person interviews has an important role to play in pain management, as it helps to build relationships between patients and medical professionals. Alternatively, when outpatients exhibited endurance, their pain worsened, and they fell into a negative cycle of poor pain control.

## Data Availability

The datasets generated and/or analyzed in this study are not publicly available, as neither the Ethics Committee nor the participants have given permission to share sensitive data, but are available from the corresponding author upon reasonable request.
